# Microwave Treatments of Cereals: Effects on Thermophysical and Parenchymal-Related Properties

**DOI:** 10.3390/foods9060711

**Published:** 2020-06-01

**Authors:** Anna Angela Barba, Carlo Naddeo, Silvestro Caputo, Gaetano Lamberti, Matteo d’Amore, Annalisa Dalmoro

**Affiliations:** 1 Dipartimento di Farmacia, Università degli Studi di Salerno, via Giovanni Paolo II, 132-84084 Fisciano SA, Italy; aabarba@unisa.it (A.A.B.); mdamore@unisa.it (M.d.A.); 2 Dipartimento di Ingegneria Industriale, Università degli Studi di Salerno, via Giovanni Paolo II, 132-84084 Fisciano SA, Italy; cnaddeo@unisa.it (C.N.); glamberti@unisa.it (G.L.); 3 Centro per la Ricerca Applicata in Agricoltura (CRAA), via G. Porzio Centro Direzionale—Isola A6, 80143 Napoli, Italy; scaputo@libro.it

**Keywords:** microwave heating, pest disinfestation, food sanitization, cereals, dielectric properties, thermal properties

## Abstract

Dielectric heating is one of the most interesting techniques for pest disinfestation. However, most of the literature works give information about the ability of microwave treatments at different power-time conditions to kill insects; less is given about the analysis of matrices structural properties and heat transport. Accordingly, the aim of this work is to investigate the effect of microwave treatments, applied for pest disinfestation, on heat transport behavior and physical/structural properties, such as water uptake capability, mineral losses, texture change, and germination capability, of most consumed cereals in human diet, such as weak wheat, durum wheat, and corn. Two different radiative treatments were performed: one in time-temperature conditions capable of inactivating the weed fauna, and the other at high temperatures of ~150 °C, simulating uncontrolled treatments. Heat transport properties were measured and showed to keep unvaried during both effective and uncontrolled microwave treatments. Instead, grain physical properties were worsened when exposed to high temperatures (reduction of germination ability and texture degradation). The achieved results, on the one hand, provide new structural and heat transport data of cereals after microwave treatments, actually not present in the literature, and on the other, they confirm the importance of correctly performing microwave treatments for an effective disinfestation without affecting matrices physical properties and nutritional features.

## 1. Introduction

Cereal grains, such as wheat, rice, and maize, are the most popular food crops in the world, and they play such an integral part in global agriculture and diet, bringing high energy content, with valuable protein, fat, and minerals. In particular, the main nutritional role of cereals is due to the high starch content which is the reserve polysaccharide, a slow release source of energy (that can be gradually used over time). When taken in the unrefined, i.e., integral, form, cereals can also be a fiber source [1]. Thus, there is a growing need to improve the preservation of large amounts of cereals supplies for human feeding, avoiding quality collapses not only in the stages of production of raw materials, but also, and especially, in the subsequent steps of handling, processing, and storage of food [2]. Improper handling, inefficient processing facilities, biodegradation due to microorganisms, and insects are the most common causes of cereals losses [3]. Storage losses can reach 50–60% due only to the technical inefficiency [4]. The storage losses are affected by two classes of factors: biotic factors (insect, pest, rodents, and fungi) and abiotic factors (temperature, humidity, and rain) [5]. In particular, among all the biotic factors, insect pests are considered the most important cause of huge losses in cereals, reducing the nutritional value with consequent risk for the operators and the consumers. Conventional techniques for disinfection and sanitization of cereals are based on chemical methods, i.e., fumigation with toxic gases, such as methyl bromide or phosphine from aluminium phosphide or calcium phosphide [6]. However, they suffer from several limitations: high costs, development of genetic resistance in the treated pests, health hazards due to toxic residues, and environmental contamination [7]. Heat treatments are common alternatives to chemical disinfestations treatments [8]; however, heat conduction and convection-based methods are limited by the relatively low thermal conductivity of foods, requiring prolonged heat exposure, therefore causing quality losses [9]. On the contrary, dielectric heating is one of the most interesting techniques of disinfestation over the last 50 years [10] for its several advantages, i.e., fast heat transfer; volumetric, selective, and uniform heating; and reduced process time, costs, and sanitization times [11,12,13]. In effect, in microwave-assisted disinfestation, exploiting the thermal effects of microwaves related to the interaction of electromagnetic fields with polar molecules, it is possible to rapidly raise the temperature of the infesting biological forms above their “lethal temperature” for their larger amount of water content respect to the dry materials (such as cereals at post-harvest conditions), which therefore are unaffected or slightly heated by microwaves [14,15,16]. For example, in recent literature, Jian and coworkers studied the difference between continuous and intermittent microwave treatment methods on wheat kernels, discovering that there was not incidence on insects mortality, set at ~75% [17]. Infested wheat grain and flour were successfully treated for disinfestation with microwave, gamma radiation, or both: the matrices showed no detectable changes in the quality of protein, fat, fiber, carbohydrates, or ash, only the germination of wheat grain was lowered after microwave treatment [2], as previously found by Vadivambal and coworkers, which also showed that milling and baking properties were kept unchanged after microwave treatment [18]. Moreover, microwave treatment at mild conditions (both low times and temperature) was proved to be able to extend the storage time of wheat flour [19]. In general, most of the literature works aim to demonstrate the possibility to kill insects by changing power-time conditions of microwave treatment [20]; less is found about the analysis of cereals structural properties and especially heat transport features.

Therefore, the aim of this work was to investigate the thermophysical and structural properties of selected cereal matrices after microwave treatments and demonstrate the importance of correctly performing the microwave process to keep intact the microbiological safety and the thermophysical properties of the irradiated cereals. The aforementioned cereal properties were checked after they were exposed to two microwave radiative protocols, as already performed in studies on legumes [16]. A multimodal cavity operating at a frequency of 2.45 GHz and a power of 1000 W have been used and two treatments were applied: one, defined “effective”, by setting the time necessary to reach a temperature higher than the lethal one, for the typical infestants in cereals, in the solid mass, and a second one, defined “uncontrolled” with a longer time of exposition to microwaves as example of not suitable thermal treatment.

## 2. Materials and Methods

### 2.1. Grain Samples

Three types of cereals seeds were selected, i.e., durum wheat (*Triticum durum* v. Creso), weak wheat (*Triticum aestivum* v. Bologna), and corn (*Zea mays* L. (NK Famoso) Cl/FAO 500–127gg). The matrices, generously supplied by the farm Improsta, Eboli (SA), Italy, were stored at room conditions and used, depending on the characterization type, as received or in milled form (fine powders were achieved by using a grinder, powders were sieved by using a 200 μm mesh size sieve).

### 2.2. Microwave Treatments

Dielectric properties, i.e., ε′, dielectric constant, accounting for how much energy is stored in the material; ε″, loss factor, measuring the overall energy dissipated in irradiated material; and, as a consequence, *D*_p_, the penetration depth, indicating the spatial distribution of the dissipative phenomena, of untreated milled grains, in bulk density conditions, were assayed adopting a network analyzer (Agilent Technologies mod ES 8753, Milano, Italy) equipped by a coaxial measuring probe (Agilent Technologies mod 85070D). The range error of the method was ~5%; measurements were performed after a calibration procedure (with three standards: air, shorting block, and water); moreover, measurement accuracy was validated using water at 20 °C. Approximately 10–12 g of milled grains (roughly 10 cm in sample thickness) was put in a glass vessel with a diameter lightly larger than that of the probe (19 mm). The probe was carefully placed on a flat sample surface without perturbing the density and avoiding air gaps. Results were reported as average values of three measures with standard deviation (SD).

Moisture content percentage (% on a wet basis, wb) of not treated powders (milled grains) was measured by the Ohaus moisture analyzer mod MB45 (Parsippany, NJ, USA).

The microwave treatment of cereals seeds was performed in a multimodal microwave cavity (LBP 210/50 Microwave Oven 2300 W, InLand, IL, USA; operative frequency: 2.45 GHz) with two integrated mode stirrers and equipped by the True-To-Power™ system, for a continuous variation of the power supply. The seeds were put in a Pyrex vessel (diameter × height: 11 cm × 6 cm), placed at the center of the cavity (nominal dimensions’ d × h × w: 53.5 × 33.0 × 25.1 cm). The penetration depth (*D*_p_) was considered as the maximum limit for the thickness of the seeds layer to be subjected to microwave treatment. A temperature between 60 and 75 °C (both surface and bulk temperature were measured by the Simpson mod. IR-10 infrared pyrometer, Milano, Italy), i.e., over the lethal temperature of typical weeds of wheat [21] and corn [22], was needed for an effective treatment. Taking into account that most species will not survive more than 1 min at 55 °C and 20 s at 65 °C [23], matrices were exposed to a microwave protocol characterized by a power of 1000 W to obtain a temperature between 60 and 75 °C at least for 20 s: they were indicated as “Effective-treated Matrices” (EM). An UnControlled Treatment (UCM) was also tested, again setting the power at 1000 W, but prolonging for three times the effective treatment time. UNtreated matrices (UN) were used as control.

### 2.3. Powder Properties

#### 2.3.1. Thermal Properties

The behavior of food to the heat was investigated by measuring volumetric heat capacity *C* (J·kg^−1^·K^−1^), thermal conductivity *K* (W·m^−1^·K^−1^), and thermal diffusivity *D* (mm^2^·s^−1^). The measure-probe of KD2 Pro, Decagon Devices was introduced in the bulks (in conditions of free and tapped) of milled UN, EM, and UCM matrices.

Bulk density (after free flowing), ρ_B_, defined as the ratio between sample mass/occupied volume without compacting, and tapped density, ρ_T_, i.e., the ratio between sample mass/occupied volume after mechanical tapping were measured on milled grains (for thermal properties measures) following the standard ASTM D7481-09.

Water contents of milled UN, EM and UCM matrices were measured in the Ohaus moisture analyzer (mod MB45). The results were reported as mean values of three measures with SD standard deviation.

#### 2.3.2. TGA and DSC

Thermogravimetric analysis (TGA) was performed in the Mettler-Toledo TGA/SDTA851 thermogravimetric analyzer (Mettler - Toledo, Milano, Italy) by increasing temperature in the range 30 to 1100 °C (heating rate: 10 °C·min^−1^) under air atmosphere (to detect ashes) or only nitrogen environment (gas flow rate of 60 mL min^−1^). Differential scanning calorimetry (DSC) was done by the METTLER TOLEDO DSC 822e thermal analyzer from 0 to 500 °C (heating rate of 5 °C·min^−1^; pure nitrogen environment was used).

TGA and DSC measurements were performed on milled samples (UN, EM, and UCM) and gave more information about the thermal stability of the analyzed cereals.

### 2.4. Grain Properties

#### 2.4.1. Size, Moisture, Density

Grain size variations (length, width, and thickness), before (UN) and after microwave heating processes (EM and UCM), before and after cooking treatments (described in Section 2.4.5), were assessed by a manual meter on several dozen randomly selected grains. The results were reported as mean values with SD standard deviation.

Bulk and tapped density determination and moisture analysis content on seeds were performed as above reported for milled grains (Section 2.3.1).

#### 2.4.2. Germination Capability

Sprouting test was conducted by placing several dozens of seeds (for UN, EM, and UCM samples) in Petri plates on well moisturized wadding, at room conditions, and moistening daily for 5 days of observation. The presence of a 0.5 cm minimum length sprout was used as sprouted grain referring. The percentage germination of seeds (defined as the percentage ratio between the germinated seeds and the total used grains), was thus assessed by measurements performed in triplicate. The germination capability was thus reported as average values with standard deviation SD.

#### 2.4.3. Water Uptake and Mineral Losses

Water uptake (or swelling tests) test was made on seeds (all the kinds of samples) to measure water absorption capability; mineral losses was assayed measuring electrolytes leaching in the soaking water [24]. Several dozen seeds were immersed in 100 mL of distilled water at ambient conditions for a maximum of 24 h. The soaked seeds were then put on paper after a certain interval of time to remove excess water, weighed and put back into the water. The swelling ratio was reported as a percentage of the absorbed water:

(1) $$swelling\;ratio\%=absorbed\;water\;\%\;=\frac{soaked\;seeds\;weight-initial\;seeds\;weight}{initial\;seeds\;weight}\times 100$$

The water absorption measurements were made in triplicate; the results were reported as mean values with SD standard deviation.

Electrolytes losses have been measured during the swelling runs through the mineral concentrations of the soaking solutions, whose conductivity increases were analyzed by the Crison Basic GLP 31 conductivity meter. The increase in the conductivity of the solution (µS/cm) was defined as the difference between the conductivity measured at time t and the water initial conductivity (at time t_0_). Conductivity measurements were performed in triplicate; the results were reported as mean values with SD standard deviation.

#### 2.4.4. Tissue Structure Analysis

To investigate eventual changes on parenchymal structure of UN, EM, and UCM samples, many of the different seeds were swollen in water for 17 h, frozen by immersion in a gel at −20 °C, and transversally cut to obtain thin films of 5 μm. Optical microscope (Leica DM-LP, 10× objective, Milano, Italy) imagines were performed with the purpose of observing the typical internal structure of cereals with packed cells filled with starch granules cemented by a solubilized protein network after immersion [25].

#### 2.4.5. Cooking Treatments and Structure Related Measurements

A typical cooking treatment, by first soaking the UN, EM, and UCM seeds in water for 17 h and then cooking them for 2 h, was applied. Grain characterization was carried out after the immersion and after cooking for 1 h, 1.5 h, and 2 h. In particular, seed water content and size variation were measured as previously reported in Section 2.4.1. Seed dimension increase % (i.e., length, width and thickness increases, %) was evaluated by

(2) $$dimension\;increase\;\%=\frac{dimension\;at\;time\;t-initial\;dimension}{initial\;dimension}\times 100$$

Moreover, penetration and compression tests were performed on cereals with the aim to evaluate eventual modifications of peel strength and tissue architecture after both swelling and cooking due to the microwave treatment. The TA.XTplus texture analyzer (Stable Micro Systems Ltd., Godalming, UK, equipped by a loading cell of 5 kg) was used; the P2N needle probe and the P/20N cylinder probe were used for penetration and compression tests, respectively. Penetration and compression force on a single UN, EM, and UCM seed, placed with the cotyledon crease parallel to the support surface, after swelling (for 17 h) and after the selected cooking intervals (1 h, 1.5 h, and 2 h), were measured setting a probe speed of 0.05 mm/s and a strain percentage of 70% in penetration tests and of 90% in compression tests. Three parameters were considered for the penetration assessment: the penetration force (kg), the peel elasticity (% strain), and the slope after peak. The penetration force, or peel hardness, was defined as the peak of the curve “force vs. strain” [26]. The peel elasticity, i.e., how much the peel resists before rupture, was given by the strain at the penetration force. The curve after the peak can be seen as a straight line, thus its slope was an index of the firmness of internal structure (higher the slope larger the compactness). The compression test was performed following the standard ASAE 5368.3 “Compression Test of Food Materials of Convex Shape”, and three important values were obtained [27]: the elastic module; the bioyield point or break point of the microstructure; and the rupture point, that is, the break point of the macrostructure.

### 2.5. Statistical Analysis

T test was used to evaluate the experimental data significance. It was applied (by Excel data sheet) to confront powder and grain measured properties. *p*-value represents the probability that differences between two measured values samples are casual or not: if *p* < 0.05, there is difference between two values (UN vs. EM or UN vs. UCM sample characteristics), on the contrary, if *p* > 0.05, the two values are comparable.

## 3. Results and Discussion

### 3.1. Microwave Treatments

Before exposing cereals seeds to microwave heating protocols, the dielectric properties of milled seeds were measured with the aim to understand matrices ability to interact with the electromagnetic field. Dielectric properties measurements are shown in the Supplementary Material Figure S1, with relevant values of moisture and bulk density for each sample. In particular, the dielectric constants, ε′, at the most common operative frequency of 2.45 GHz, of both durum wheat and corn were of 2.39 (moisture content 6.3% wb) and 2.6 (moisture content 10.5% wb), respectively, close to the values found in [28] for milled similar products with the same moisture (for hard red winter wheat, ε′ ~2.3 with a moisture of ~6.5%; for corn, ε′ ~2.8 with a moisture of ~10.5%). Moreover, the value of ε′ = 2.9 at 2.45 GHz for weak wheat was similar to the ε′ = 2.84 found in similar conditions by [29]. Thus, a volumetric heating was assured using a seeds layer of 4 cm (the penetration depth, *Dp*, calculated from them by the Equation (2), was roughly 7 cm). Surface temperatures were measured during both effective (EM) and uncontrolled (UCM) treatments (Supplementary Material Figure S2): the effective treatment (EM) was performed up to the attainment of the maximum temperature of 75 °C for 105 s, 75 s, and 100 s, the UCM one was prolonged up to 315 s, 225 s, and 300 s, reaching a final temperature of 150 °C, respectively, for weak wheat, durum wheat, and corn. During the microwave treatment, the temperatures reached by pests, which have a larger content of water, will be higher than the temperatures of matrices, allowing the treatment to kill them.

### 3.2. Powder Properties

**Figure 1:**
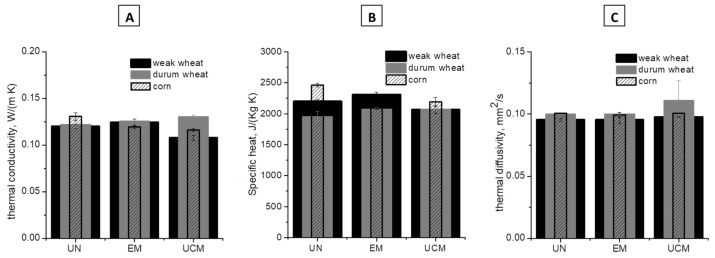
Thermal properties ((**A**) thermal conductivity, (**B**) specific heat, and (**C**) thermal diffusivity) in tapped density conditions of untreated (UN), effective treated (EM), and uncontrolled treated (UCM), in black column, powdered weak wheat (moisture—tapped density: UN = 10.6 ± 0.3 wb%–0.74 ± 0.01 g/mL; EM = 10.3 ± 0.4 wb%–0.77 ± 0.00 g/mL; UCM = 6.3 ± 0.3 wb%–0.70 ± 0.02 g/mL), in gray column, durum wheat (moisture—tapped density: UN = 6.3 ± 0.4 wb%–0.75 ± 0.00 g/mL; EM = 8.0 ± 0.4 wb%–0.73 ± 0.01 g/mL; UCM = 7.3 ± 0.3 wb%–0.74 ± 0.00 g/mL), in striped column, corn (moisture–tapped density: UN = 10.5 ± 0.2 wb%–0.74 ± 0.00 g/mL; EM = 9.4 ± 0.3 wb%–0.69 ± 0.02 g/mL; UCM = 6.0 ± 0.1 wb%–0.67 ± 0.00 g/mL).

Thermal properties are fundamental to predict energy transfer rates in foods, thus they affect food processes, such as cooking or drying, storage, and final quality of foods [30]. The three thermal characteristics—specific heat and thermal diffusivity and conductivity—in tapped density conditions for UN, EM, and UCM, three types of cereals, are shown in Figure 1 (their values in bulk conditions were not shown because were similar before, UN, and after the two microwave heating, EM and UCM).

In the literature, it was found that thermal conductivity (Figure 1A) increased with increasing both moisture and density [31,32]. In effect, for both weak wheat and corn, moisture and tapped density after EM treatment were kept unchanged in the range 10 to 11% and 0.7 to 0.77 g/mL respectively, thus thermal conductivity values were similar between UN and EM samples. Instead, after UCM treatment, both weak wheat and corn were subjected to the lowering of moisture to ~6% and of tapped density, especially for corn to 0.67 g/mL, causing a visible reduction of thermal conductivity (comparison between UN and UCM thermal conductivity gave *p* < 0.05). For durum wheat, both moisture and tapped density were comparable among UN, EM, and UCM samples and, as a consequence, also the relevant values of thermal conductivity (UN vs. EM and UN vs. UCM gave *p* > 0.05). The behavior of specific heat was similar to that of thermal conductivity (Figure 1B). Values of specific heat are in line with literature values for cereals [33]. Thermal diffusivity (Figure 1C) was proven to decrease by increasing the moisture content and decreasing density [32]. Thus, the compensation of these two effects in UCM samples of both weak wheat and corn (i.e., decreasing of moisture and of tapped density, which should increase and decrease respectively the thermal diffusivity) kept essentially unchanged the thermal diffusivities for the UN, EM and UCM samples (*p* > 0.05). Moreover, a low number of literature works deals with thermal properties of cereals at conditions of density and moisture similar to ours. However, the authors of [34] found a thermal diffusivity in the range 0.099 to 0.104 mm^2^/s for wheat flour with around 12% of moisture and a density of 0.784 g/mL. Moreover, the authors of [35] found a similar value of diffusivity. In similar conditions of moisture (both UN and EM of about 10–11%) and density (tapped density of both UN and EM in the range 0.74 to 0.77 g/mL), we measured a thermal diffusivity of 0.095. TGA analysis gave information about moisture and ashes (Supplementary Material Figure S3A), which were similar for the three analyzed samples (UN, EM, and UCM). In effect, the initial weight losses were of ~7%, values confirmed by moisture analysis performed by both Ohaus instrument (UN = 6.3 ± 0.4% wb; EM = 8.0 ± 0.4% wb; UCM = 7.3 ± 0.3% wb) and literature [36]. The second important mass loss resulting from thermal decomposition occurred between temperatures of 200 °C and 800 °C [37] and distinct mass loss occurred between 200 and 400 °C due to cellulose devolatization, which is a dominant phenomenon in comparison to hemicellulose and lignin pyrolysis [38]. At the end of pyrolysis, the remained ash content was observed to be of ~3–5% for all the three samples.

Also DSC thermograms were similar for all the analyzed matrices (Supplementary Material Figure S3B): they showed an endothermic peak, which starts before 100 °C, due to moisture losses; successively an exothermic peak nearby 300 °C and 350 °C, indicators of degradation phenomena of the seed coat cellulose [39].

### 3.3. Grain Properties

The grain size of the three analyzed matrices was not affected by the effective (EM) or by the uncontrolled (UCM) microwave heating (data not shown). By contrast, the three matrices were subjected to a reduction in bulk and tapped density after the uncontrolled treatment (*p* < 0.05 if compared with UN samples), as shown in Table 1.

This effect could be a result of the expansion and cracking of endosperm due to the higher temperatures reached in UCM samples. In effect, density is frequently used as an indirect indicator of the corneous endosperm content in corn because the corneous endosperm is very dense, while floury endosperm is full of microcracks or empty spaces, thus less dense [40]. Quality of weak wheat was the most affected after UCM treatments, due to a reduction in both moisture and weight (*p* < 0.05), corn had only reduced moisture (*p* < 0.05), instead durum wheat kept unchanged both (*p* > 0.05). Moreover, the look of cereals matrices was prejudiced by uncontrolled microwave heating that caused a browning because of the high temperatures reached in UCM samples.

The germination capability was an indication of protein denaturation and starch recovery [41] and it was largely influenced by microwave treatment (Table 2). In particular, it is observed that germination capability decreased as exposure time increased [22,42]. Thus, the effective treatment (EM) caused a visible reduction in germination especially for corn (with an already low level of germination in untreated samples UN because corn germination needs a minimum room temperature of 12 °C compared to wheat that starts to germinate also at 2–4 °C), but also for weak wheat, having EM treatment times of respectively 100 s and 105 s. EM durum wheat kept intact its germination rate for the lower treatment time of 75 s. Then, the uncontrolled treatment (UCM) caused for all the three matrices a lack of germination due the disruption of cell content integrity for the very high temperatures, as already observed by densities reduction.

The analysis of parenchymal internal structure confirmed the germination tests results.

**Figure 2:**
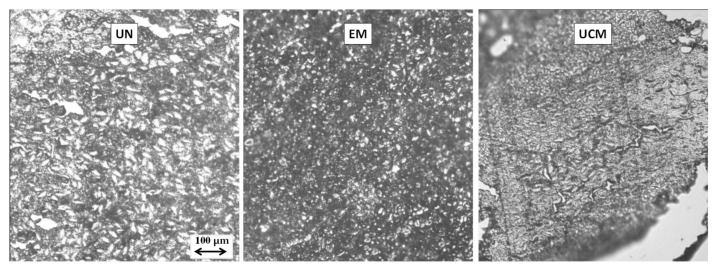

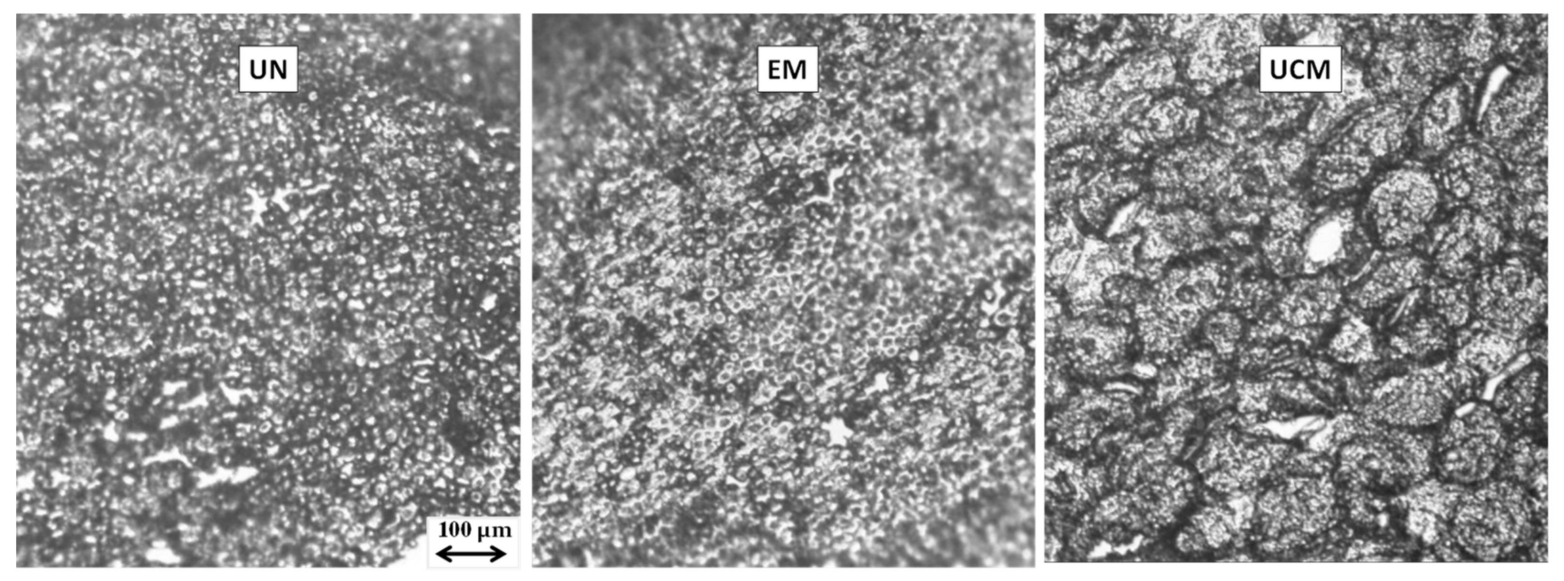
Images performed at optical microscope (10× objective) of transversal layers of 5 μm, cut from UN, EM, and UCM durum wheat grains (**up**) and corn grains (**down**), first immersed in water for 17 h and then frozen at −20 °C.

**Figure 3:**
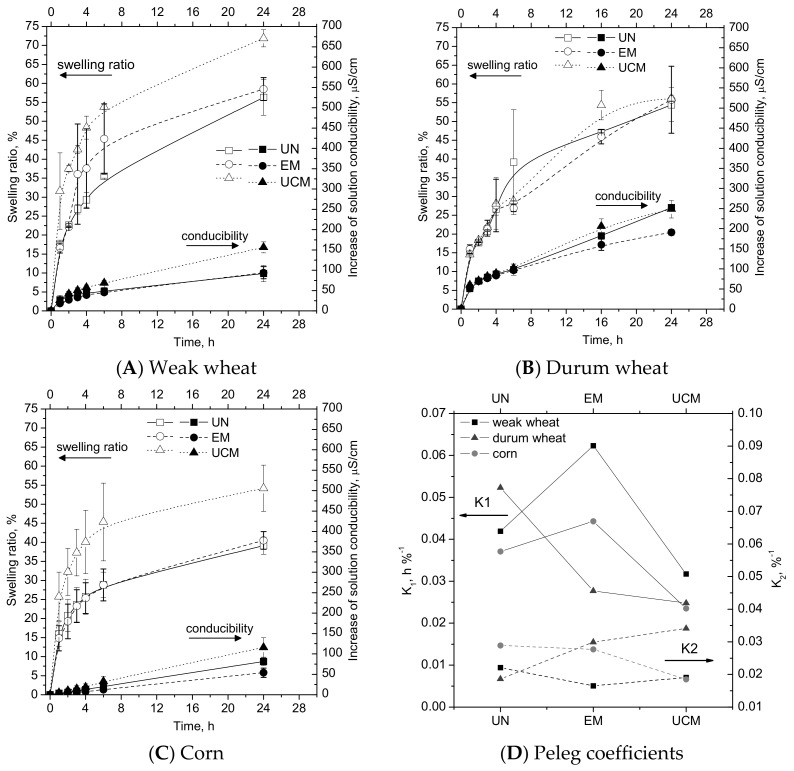
Percentage of swelling ratio (left side, open symbols) and solution conductibility increase (right side, closed symbols) for UN (squares), EM (circles), and UCM (triangles) grains of weak wheat (**A**), durum wheat (**B**), and corn (**C**). (**D**) Peleg coefficients (*K*_1_ left side, *K*_2_ right side) for UN, EM and UCM weak wheat (squares), durum wheat (triangles), and corn (circles).

As an example, durum wheat and corn pictures are shown in Figure 2 (up and down, respectively): the endosperm of both UN and EM wheat and corn seeds showed a compact structure of densely packed cells filled with starch granules firmly cemented by a protein network, always solubilized after soaking. In UCM wheat and corn, denatured proteins covering a more compact structure made of highly gelatinized and deformed granules within the kernel were visible for the too much high temperatures of the treatment, as already observed by [43] for corn flour. In particular, intra-grain cracks were shown in UCM samples of durum wheat (Figure 2, up). It was shown that higher gluten protein seeds (wheat) seem to tolerate higher levels of starch damage [44], instead in UCM samples of corn (Figure 2, down), the disappearance of the starch granules (that are present in higher percentage in corn), caused a more porous structure. This behavior affected swelling properties (Figure 3).

In particular, all the three durum wheat samples (UN, EM, and UCM) had a similar behavior (Figure 3B), EM for the low treatment time and UCM perhaps for the two opposing effects of a more compact structure (high gelatinization) and the presence of cracks, that make its swelling and mineral leaching behavior similar to UN and EM. On the contrary, UCM samples of weak wheat (Figure 3A) and corn (Figure 3C) absorbed water and lost minerals more rapidly than EM and UN ones, thus confirming the not more compact structure of UCM samples after high temperature treatments. Using the Peleg equation (it was used to regress the water content vs. the soaking time: $\frac{t}{M-M_{0}}=K_{1}+K_{2}\cdot t$, in which *t* is the time (h); *M* and *M*_0_ are the water content and the initial water content, respectively (% on dry basis); *K*_1_ indicates the Peleg rate constant (h %^−1^), correlated to the mass transfer rate, i.e., lower the *K*_1_, the higher the initial water absorption rate; and *K*_2_ represents the Peleg capacity constant (%^−1^), correlated to the maximum moisture absorption capability, thus the lower the *K*_2_, the higher the water absorption capacity [16,25]), it can be seen that the constant *K*_1_ for all the three matrices (Figure 3D) was lower for all the UCM samples, therefore the initial water uptake rate was higher for them. Instead *K*_2_, i.e., the water absorption capacity, was similar for untreated and treated samples. Finally it must be noticed that, this starch damage affected not only water absorption, but also handling properties of the dough, sugar production, slackening during fermentation, loaf volume and crumb tenderness [44].

**Figure 4:**
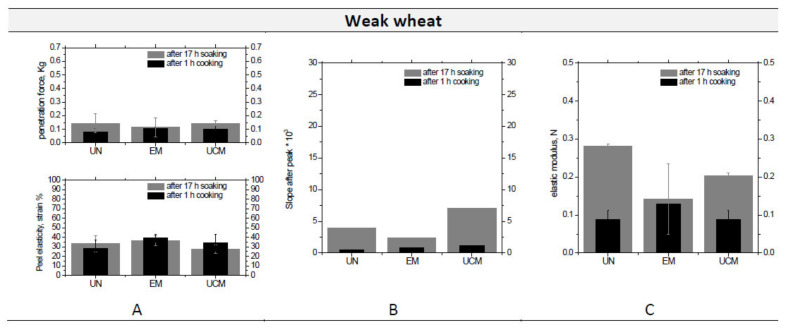

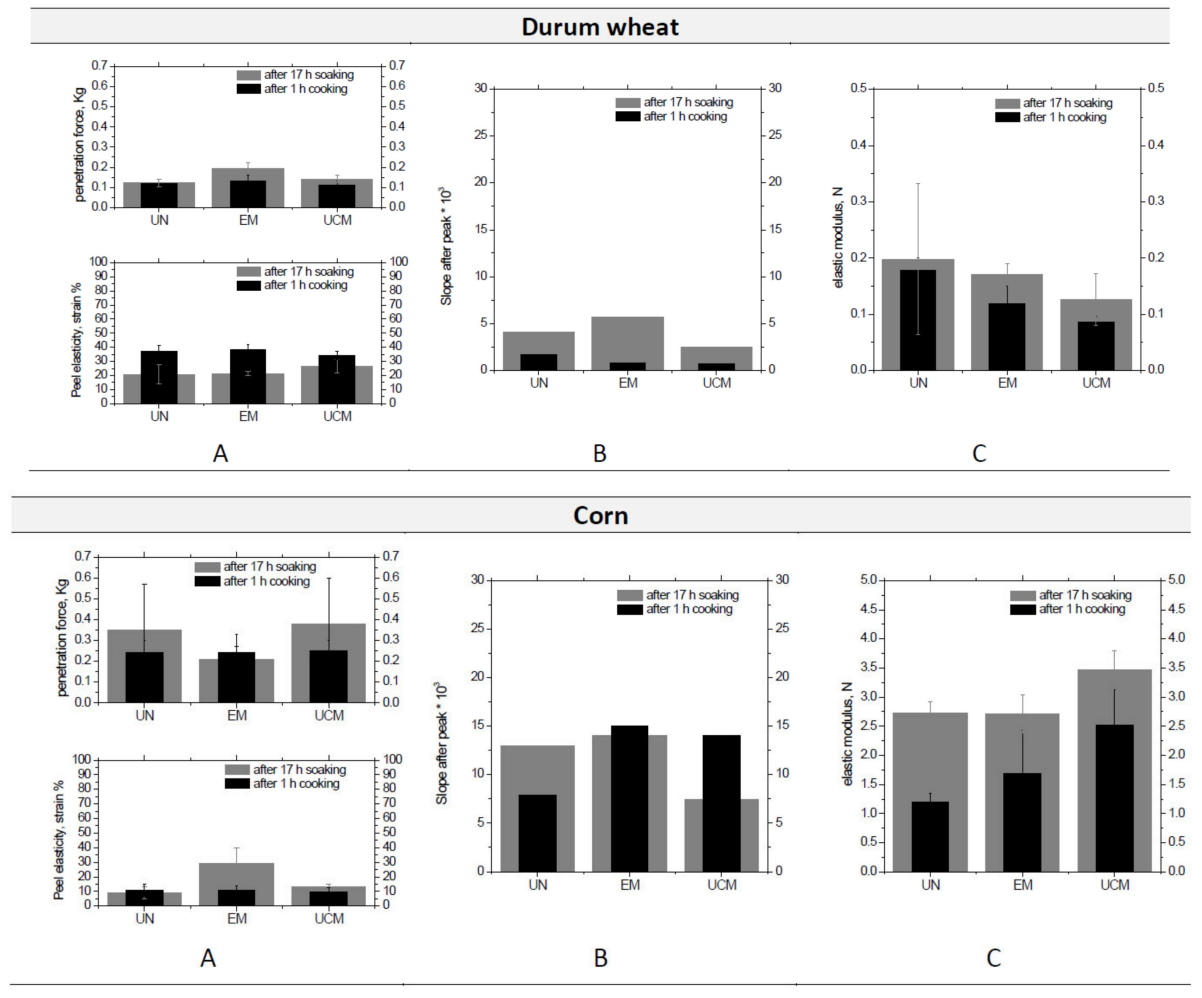
Penetration and compression tests results for the three analyzed matrices: comparison of the penetration force and the relevant strain % (**A**) and the slope after the peak (**B**) for the penetration test, and of the elastic modulus (**C**) for the compression test between samples after 17 h swelling and 1 h cooking.

Grain properties, in particular moisture increase and size variation, were also evaluated during the stages of common cooking preparation treatment: after a first pretreatment of swelling in water for 17 h, and after cooking for 1 h, 1.5 h and 2 h (even if the grains of both weak wheat and corn lost their shape after 1.5 h of cooking). Moisture content change (data not reported) behavior was similar for the untreated and treated samples of the three matrices (as the previously deduced by the comparable values of *K*_2_ indicating an analogous water up-take capability), i.e., the moisture % increased up to 1.5 h of cooking and then kept constant at ~60–70%. Cooking influenced very little the length of the three samples, but largely both their width and thickness (data not shown), confirming the radial entrance of water. Cooking obviously modified peel strength and internal structure of the three matrices, as visible by penetration and compression tests. Results from penetration and compression tests between grains only soaked for 17 h (first step of cooking) and grains subjected to 1 h of cooking (after the soaking, thus second step of cooking) were compared in Figure 4. It is evident that the cooking process (after 1 h) caused: a light reduction of the penetration force for all the three analyzed matrices and a light increase (for weak and durum wheat) or a constancy (for corn) of the corresponding peel elasticity (strain % at the penetration force) (Figure 4A) and of the slope after the curve (Figure 4B). The elastic modulus (Figure 4C) decreased for all the three matrices after 1 h of cooking. Statistically, UN, EM, and UCM had mostly the same behavior at penetration tests; however, in the compression tests, the elastic modulus was comparable for UN, EM, and UCM weak wheat samples, but it had an opposite behavior for the other two matrices. In particular, the elastic modulus was lower for UCM samples of durum wheat (compared to both UN and EM, especially to UN) at both 17 h soaking and 1 h cooking, confirming the increased elasticity after uncontrolled heating (the compact cells filled with starch granules strongly sticked by a protein matrix observed by optical microscope). The UCM corn samples had, on the contrary, an elastic modulus higher than UN, confirming the formation of a more porous internal structure (Figure 4, down).

## 4. Conclusions

In this work, the thermo-physical and structural properties of three cereal matrices—weak wheat, durum wheat, and corn—were investigated after microwaves treatments, intended for pest disinfestation. In particular, two microwave protocols were developed and applied: an effective microwave treatment (EM), ensuring a temperature in cereal mass of ~60–75 °C for at least 20 s (time-temperature couple able to eliminate insects, as observed in literature) and a more drastic, named uncontrolled, microwave treatment (UCM), performed by prolonging the irradiation time up to three times that used for effective treatment. The application of the two different microwave protocols to cereal grains of durum wheat, weak wheat, and corn caused no substantial modification on the heat transport properties, instead drastic treatment (UCM) compromised the nutritional (loss of minerals), sensorial (fragile texture) and germination (poorer amyliferous reserve) features. The novelty of this work is thus found in providing new structural and heat transport data of cereals after microwave treatments; moreover, it was also demonstrated that only suitable microwave treatments can guarantee both effective sanitation and maintenance of the nutritional and sensory properties of cereal matrices.

## Figures and Tables

**Table 1:** Moisture content, bulk density, and tapped density for untreated (UN), effective treated (EM), and uncontrolled treated (UCM) seeds of weak wheat, durum wheat, and corn (standard deviation (SD)).

Weak Wheat	UN	EM	UCM
Moisture, % wb ± SD	1.36 ± 0.01	1.29 ± 0.19	0.55 ± 0.01
Bulk (tapped) density, g/mL ± SD	0.74 ± 0.01	0.73 ± 0.02	0.55 ± 0.00
	(0.77 ± 0.01)	(0.76 ± 0.03)	(0.57 ± 0.01)
**Durum wheat**	**UN**	**EM**	**UCM**
Moisture, % wb ± SD	1.24 ± 0.19	1.16 ± 0.09	0.96 ± 0.09
Bulk (tapped) density, g/mL ± SD	0.80 ± 0.01	0.79 ± 0.01	0.59 ± 0.03
	(0.84 ± 0.01)	(0.83 ± 0.01)	(0.62 ± 0.01)
**Corn**	**UN**	**EM**	**UCM**
Moisture, % wb ± SD	0.83 ± 0.19	0.44 ± 0.11	0.23 ± 0.08
Bulk (tapped) density, g/mL ± SD	0.65 ± 0.01	0.60 ± 0.01	0.55 ± 0.01
	(0.65 ± 0.01)	(0.60 ± 0.01)	(0.56 ± 0.03)

**Table 2:** Germination percentage (±SD) for UN, EM, and UCM samples of weak wheat, durum wheat, and corn.

Weak Wheat		
UN	EM	UCM
100 ± 0	59 ± 2	0
**Durum wheat**		
**UN**	**EM**	**UCM**
88 ± 2	93 ± 3	0
**Corn**		
**UN**	**EM**	**UCM**
30 ± 5	7 ± 7	0

## Supplementary Materials

The following are available online at https://www.mdpi.com/2304-8158/9/6/711/s1, Figure S1: Permittivity, i.e. dielectric constant (Re) and loss factor (Im) for milled weak wheat (closed and open squares, respectively), durum wheat (closed and open triangles, respectively), corn (closed and open circles, respectively), and moisture contents; Figure S2: Behavior of surface temperature with time for weak wheat (squares), durum wheat (triangles), corn (circles), initial moisture contents, and both effective (EM) and uncontrolled (UCM) treatment times; Figure S3: TGA curves in presence of air in A) and DSC curves in B) for UN (black), EM (dark gray), and UCM (light gray).

## References

[1] Cappelli P., Vannucchi V. (1990). Chimica Degli Alimenti: Conservazione e Trasformazione.

[2] El-Naggar S., Mikhaiel A. (2011). Disinfestation of stored wheat grain and flour using gamma rays and microwave heating. J. Stored Prod. Res..

[3] García-Mosqueda C., Salas-Araiza M.D., Ceron-Garcia A., Estrada-García H.J., Rojas-Laguna R., Sosa-Morales M.E. (2019). Microwave heating as a post-harvest treatment for white corn (*Zea mays*) against Sitotroga cerealella. J. Microw. Power Electromagn. Energy.

[4] Kumar D., Kalita P.K. (2017). Reducing Postharvest Losses during Storage of Grain Crops to Strengthen Food Security in Developing Countries. Foods.

[5] Abedin M.Z., Rahman M.Z., Mia I.A.M., Rahman K.M.M. (2012). In-store losses of rice and ways of reducing such losses at farmers’ level: An assessment in selected regions of Bangladesh. J. Bangladesh Agric. Univ..

[6] Shaaya E., Kostjukovski M., Eilberg J., Sukprakarn C. (1997). Plant oils as fumigants and contact insecticides for the control of stored-product insects. J. Stored Prod. Res..

[7] Tapondjou L., Adler C., Bouda H., Fontem D. (2002). Efficacy of powder and essential oil from Chenopodium ambrosioides leaves as post-harvest grain protectants against six-stored product beetles. J. Stored Prod. Res..

[8] Ben-Lalli A., Méot J.-M., Collignan A., Bohuon P. (2011). Modelling heat-disinfestation of dried fruits on “biological model” larvae Ephestia kuehniella (Zeller). Food Res. Int..

[9] Gamage T., Sanguansri P., Swiergon P., Eelkema M., Wyatt P., Leach P., Alexander D., Knoerzer K. (2015). Continuous combined microwave and hot air treatment of apples for fruit fly (*Bactrocera tryoni* and *B. jarvisi*) disinfestation. Innov. Food Sci. Emerg. Technol..

[10] Webber H.H., Wagner R.P., Pearson A.G. (1946). High-Frequency Electric Fields as Lethal Agents for Insects. J. Econ. Èntomol..

[11] Angela A., Damore M. (2012). Relevance of Dielectric Properties in Microwave Assisted Processes.

[12] Chandrasekaran S., Ramanathan S., Basak T. (2013). Microwave food processing—A review. Food Res. Int..

[13] Nasrollahzadeh F., Varidi M., Koocheki A., Hadizadeh F. (2017). Effect of microwave and conventional heating on structural, functional and antioxidant properties of bovine serum albumin-maltodextrin conjugates through Maillard reaction. Food Res. Int..

[14] Yadav D.N., Anand T., Sharma M., Gupta R.K. (2012). Microwave technology for disinfestation of cereals and pulses: An overview. J. Food Sci. Technol..

[15] Los A., Ziuzina D., Bourke P. (2018). Current and Future Technologies for Microbiological Decontamination of Cereal Grains. J. Food Sci..

[16] Dalmoro A., Naddeo C., Caputo S., Lamberti G., Guadagno L., D’Amore M., Barba A. (2018). On the relevance of thermophysical characterization in the microwave treatment of legumes. Food Funct..

[17] Jian F., Jayas D.S., White N.D., Fields P., Howe N. (2015). An evaluation of insect expulsion from wheat samples by microwave treatment for disinfestation. Biosyst. Eng..

[18] Vadivambal R., Jayas D., White N. (2007). Wheat disinfestation using microwave energy. J. Stored Prod. Res..

[19] Qu C., Wang H., Liu S., Wang F., Liu C. (2017). Effects of microwave heating of wheat on its functional properties and accelerated storage. J. Food Sci. Technol..

[20] Rifna E., Singh S., Chakraborty S., Dwivedi M. (2019). Effect of thermal and non-thermal techniques for microbial safety in food powder: Recent advances. Food Res. Int..

[21] Hamid M.A.K., Boulanger R.J. (1969). A New Method for the Control of Moisture and Insect Infestations of Grain by Microwave Power. J. Microw. Power.

[22] Vadivambal R., Deji O., Jayas D., White N. (2010). Disinfestation of stored corn using microwave energy. Agric. Biol. J. N. Am..

[23] Fields P.G. (1992). The control of stored-product insects and mites with extreme temperatures. J. Stored Prod. Res..

[24] Berrios J.D.J., Swanson B.G., Cheong W.A. (1999). Physico-chemical characterization of stored black beans (*Phaseolus vulgaris* L.). Food Res. Int..

[25] Bhatty R.S. (1990). Cooking quality of lentils: The role of structure and composition of cell walls. J. Agric. Food Chem..

[26] Lee C.M., Chung K.H. (1989). Analysis of Surimi Gel Properties by Compression and Penetration Tests. J. Texture Stud..

[27] Voicu G., Tudosie E.-M., Ungureanu N., Constantin G.-A. (2013). Some mechanical characteristics of wheat seeds obtained by uniaxial compression tests. Univ. Politeh. Buch. Sci. Bull. D.

[28] Nelson S.O. (1994). Measurement of microwave dielectric properties of particulate materials. J. Food Eng..

[29] Torrealba R., Sosa-Morales M.E., Olvera-Cervantes J.L., Corona-Chavez A. (2015). Dielectric properties of cereals at frequencies useful for processes with microwave heating. J. Food Sci. Technol..

[30] Sweat V.E. (1986). Thermal properties of foods. Engineering Properties of Foods.

[31] Božiková M. (2012). Thermophysical parameters of corn and wheat flour. Res. Agric. Eng..

[32] Mahapatra A.K., Lan Y., Harris D. (2011). Influence of Moisture Content and Temperature on Thermal Conductivity and Thermal Diffusivity of Rice Flours. Int. J. Food Prop..

[33] Kaletunc G. (2007). Prediction of specific heat of cereal flours: A quantitative empirical correlation. J. Food Eng..

[34] Magee T., Bransburg T. (1995). Measurement of thermal diffusivity of potato, malt bread and wheat flour. J. Food Eng..

[35] Kostaropoulos A., Saravacos G. (1997). Thermal diffusivity of granular and porous foods at low moisture content. J. Food Eng..

[36] Ergudenler A., Ghaly A.E. (1992). Determination of reaction kinetics of wheat straw using thermogravimetric analysis. Appl. Biochem. Biotechnol..

[37] Ross K., Godfrey D. (2012). Effect of extractives on the thermal decomposition of wheat, triticale, and flax crop residues: A kinetic study. Int.J. Biomass Renew..

[38] Dizaji H.B., Dizaji F.F., Bidabadi M. (2014). Determining thermo-kinetic constants in order to classify explosivity of foodstuffs. Combust. Explos. Shock Waves.

[39] Draman S.F.S., Daik R., Latif F.A., El-Sheikh S.M. (2013). Characterization and Thermal Decomposition Kinetics of Kapok (*Ceiba pentandra* L.)—Based Cellulose. Bioresources.

[40] Kirleis A., Stroshine R. (1990). Effects of hardness and drying air temperature on breakage susceptibility and dry-milling characteristics of yellow dent corn. Cereal. Chem..

[41] Gursoy S., Choudhary R., Watson D.G. (2013). Microwave drying kinetics and quality characteristics of corn. Int.J. Agric. Biol. Eng..

[42] Ragha L., Mishra S., Ramachandran V., Bhatia M.S. (2011). Effects of Low-Power Microwave Fields on Seed Germination and Growth Rate. J. Electromagn. Anal. Appl..

[43] Roman L., Martinez M.M., Rosell C.M., Gómez M. (2015). Effect of Microwave Treatment on Physicochemical Properties of Maize Flour. Food Bioprocess. Technol..

[44] Błaszczak W., Gralik J., Klockiewicz-Kamińska E., Fornal J., Warchalewski J.R. (2002). Effect of *γ*-radiation and microwave heating on endosperm microstructure in relation to some technological properties of wheat grain. Food/Nahrung.

