# Supplementary material for: Microwave Treatments of Cereals: Effects on Thermophysical and Parenchymal-Related Properties
Source: Foods. 2020 Jun 1;9(6):711. doi: 10.3390/foods9060711 (PMC7353526; doi:10.3390/foods9060711)
Supplement: Supplementary file 1 [file foods-09-00711-s001.pdf]

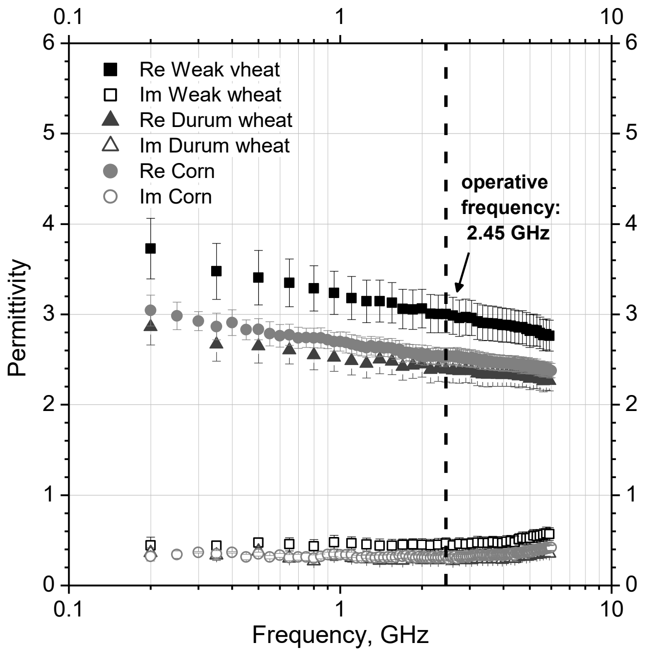

	Weak wheat	Durum wheat	Corn
Moisture, wb% ± SD	10.6±0.3	6.3±0.4	10.5±0.2
Bulk density, kg/m^3 ± SD	570 ± 6	626 ± 6	527 ± 12

Suppl Mat Figure 1. Permittivity, i.e. dielectric constant (Re) and loss factor (Im) for milled weak wheat (closed and open squares, respectively), durum wheat (closed and open triangles, respectively), corn (closed and open circles, respectively), and moisture contents

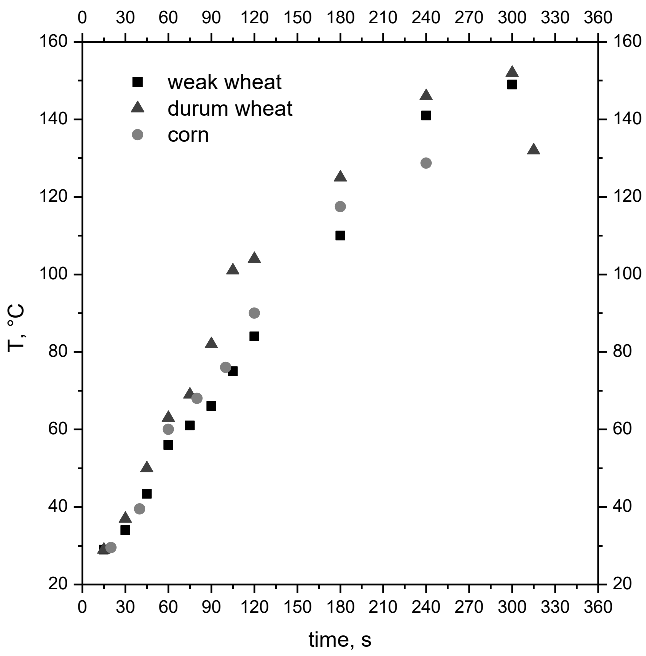

	Weak wheat	Durum wheat	Corn
Moisture, wb% ± SD	1.36 ± 0.01	1.24 ± 0.19	0.83 ± 0.19
EM times, s	105	75	100
UCM times, s	315	225	300

Suppl Mat Figure 2. Behavior of surface temperature with time for weak wheat (squares), durum wheat (triangles), corn (circles), initial moisture contents, and both effective (EM) and uncontrolled (UCM) treatment times

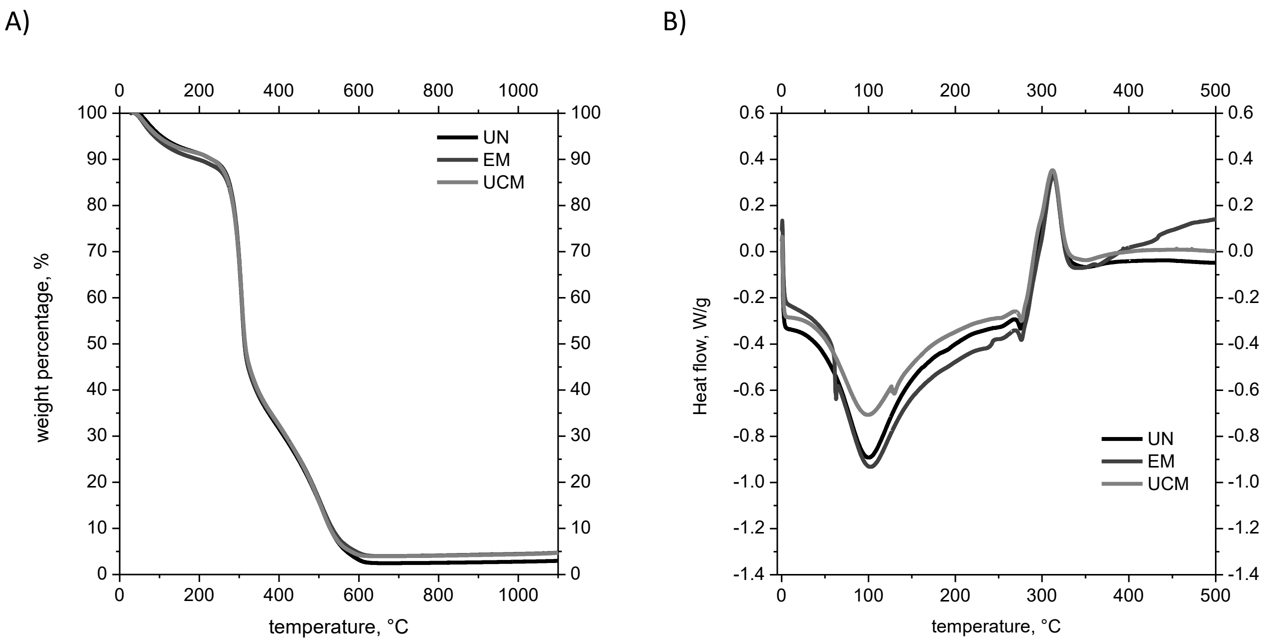

Suppl Mat Figure 3. TGA curves in presence of air in A) and DSC curves in B) for UN (black), EM (dark gray), and UCM (light gray) powdered samples of durum wheat
